# Development and evaluation of an e-learning program on drug-related problems for community pharmacists

**DOI:** 10.1007/s11096-026-02111-5

**Published:** 2026-03-25

**Authors:** Cathrin J. Vogt, Alesia Reuther, Viktoria S. Wurmbach, Marina Weißenborn, Janina A. Bittmann, Katharina Wien, Anette Lampert, Emilia Maria Boček Eknes, Patrick Schäfer, Hanna M. Seidling

**Affiliations:** 1 Medical Faculty Heidelberg/Heidelberg University Hospital, Internal Medicine IX—Department of Clinical Pharmacology and Pharmacoepidemiology, Cooperation Unit Clinical Pharmacy, Heidelberg University, Im Neuenheimer Feld 410, 69120 Heidelberg, Germany; 2 Centre for Pharmacy, University Bergen, Bergen, Norway; 3 Chamber of Pharmacists Baden-Wurttemberg, Stuttgart, Germany

**Keywords:** Continuing education, Community pharmacy, Drug-related problems, e-learning

## Abstract

**Introduction:**

Community pharmacists (CPs) are pivotal in the management of drug-related problems (DRPs), yet a need for additional training has been recognized.

**Aim:**

The aim was to develop and evaluate an e-learning program to enhance pharmacists’ ability to detect and manage DRPs.

**Method:**

The e-learning program provided a self-paced learning experience with 10 modules, each focusing on a DRP. The DRPs were chosen as part of a previous Delphi consensus study. The development team, consisting of researchers, clinical pharmacists, and CPs, considered DRPs applicable if they occurred frequently and could be solved in a pharmacy. Moreover, a predefined checklist was established to guide the development of new modules and specify quality standards. Each module was structured into an educational phase (tutorials, pre- and post-knowledge quizzes, in-depth exercises), and a practical phase (documentation of anonymized patient encounters). After completing all modules, participants took a final comprehensive quiz with 2 questions per module (in total 20 questions). The program was evaluated on multiple levels: pharmacists’ knowledge change, how they implemented this knowledge in practice, and their feedback. Data were analyzed descriptively. Comparison between the quizzes were calculated using the Wilcoxon test.

**Results:**

A total of 203 pharmacists registered to participate in the study, with participation declining across modules (184 participants completed Module 1 pre-knowledge quiz vs. 119 in Module 10). Across all modules, a significant knowledge increase was observed when comparing pre- and post-knowledge-quizzes (*p* < 0.001). Knowledge levels remained high in the final quiz (on average 1.63 ± 0.61 correct answers). Participants documented 13,778 patient encounters, thereof 5073 encounters with at least one DRP. According to the participants, most of the DRPs (90.1%, n = 4550) could be resolved. A total of 1137 feedbacks were received. Overall, feedback was positive, with participants highlighting improved abilities to engage with patients (85.2%, n = 969) and to identify DRPs (82.0%, n = 629).

**Conclusion:**

By bridging the gap between theoretical learning and practical application, this e-learning program improves pharmacists’ skills in DRP management.

**Supplementary Information:**

The online version contains supplementary material available at 10.1007/s11096-026-02111-5.

## Impact statements

- A self-paced e-learning program consisting of ten modules on common DRPs in pharmacy was developed. During the educational phase, participants completed quizzes, tutorials and in-depth exercises to strengthen their theoretical knowledge.
- To translate this knowledge into practice, participants documented real patient encounters, identified DRPs, and recorded the interventions they implemented to address them.
- By participating in this program, CPs successfully increased their knowledge and felt more confident in managing DRPs and counselling patients, which ultimately may improve patients’ health outcomes.

## Introduction

Drug-related problems (DRPs) are obstacles to effective and safe medication therapy [1] and are particularly common in patients with polypharmacy [2, 3]. Once a DRP has been identified, it should be classified into distinct categories based on the type of problem, its cause, and the intervention undertaken using a classification system, such as that developed by the Pharmaceutical Care Network Europe (PCNE) [4].

As the prevalence of polypharmacy increases [5–7], managing DRPs is becoming more challenging. Community pharmacists (CPs) can play a crucial role in the management of DRPs [8–13], i.e., the identification and solution of DRPs. However, barriers to DRP management include inconsistent IT infrastructure, variability in pharmacist training, and time constraints [11]. Moreover, a training need has been recognized [14–16] with an emphasis on prioritized learning content and reinforcement of newly acquired knowledge in practice [17]. Such targeted training can significantly enhance CPs' ability to identify [18] and resolve DRPs in patients [19]. Despite pharmacists value continuing education, they face various barriers to participation such as time constraints [20]. Although digital solutions may allow flexible time management, in the past, pharmacists preferred in-person over digital trainings [20]. Technical issues further decreased motivation for digital training [21]. However, the gain of knowledge in e-learning was similar to in-person training [22]. It may therefore be postulated that acceptance increases with higher usability of the systems.

To evaluate a training program’s effectiveness, the Kirkpatrick’s 4-level model can be used [23, 24]: Level 1 measures participant satisfaction; Level 2 assesses the extent to which participants have gained knowledge; Level 3 evaluates whether participants apply this knowledge in practice; and Level 4 examines higher-level outcomes, such as health outcomes.

### Aim

The aim was to develop a user-friendly e-learning training program supporting CPs in DRP management and evaluate its effectiveness in a pilot-test.

## Method

To evaluate the program, 3 levels of the Kirkpatrick model were assessed: (1) participants’ feedback on their satisfaction with the program, (2) change in their knowledge, and (3) their ability to apply this knowledge in practice. In collaboration with the Baden-Wurttemberg Chamber of Pharmacists, an e-learning-based training program was developed. This manuscript follows the Defined Criteria To Report INnovations in Education (DoCTRINE) reporting guideline [25] (Supplement A).

### Participants and recruitment measures

The chamber of pharmacists Baden-Wuerttemberg invited all licensed CPs working in a community pharmacy in the chamber district of Baden-Wurttemberg via mail in summer 2017. Data were collected from September 2017 to November 2018. Due to the exploratory character of the study, no sample size was calculated, and the planned number of a minimum of 50 participants was based only on feasibility considerations.

Eligible were all CPs working regularly in a community pharmacy; no additional restrictions were applied, as the program targeted CPs across all experience levels. They had to register (and thereby consent) and provide contact information and current working position in the pharmacy (employment, branch management, or ownership). Enrolled participants were assigned a pseudonym for data collection in the quizzes and the documentation forms. Feedback was collected anonymously to ensure confidentiality.

### Structure of the e-learning program

The program comprised 10 two-phased modules, each addressing one DRP (Table 1). Relevant DRPs were extracted from a literature search on DRPs identified in community pharmacies, and then prioritized using a Delphi-consensus with five pharmacists and physicians [26]. The study team selected the final set of DRPs. DRPs had to be frequent and resolvable in community pharmacy practice without physician contact.

**Table 1:** Overview of the 10 e-learning modules

Module No	Drug-related problem	Main learning subjects of the module	Content of the tutorial
1	Application of eye drops	Application skills	Correct application of eye drops
			Canthal application
			Recommendations for practice
			Start a conversation and detect problems
2	Interaction of polyvalent cations with chelating agents	Time of application, drug-drug- and drug-food- interactions	Explanation of interaction’s mechanism and impact
			When is action required
			Recommendations for practice
			Challenges
			Start a conversation and detect problems
3	Time of administration of proton pump inhibitors (PPIs)	Time of application	Prevalence and indications of PPIs
			Common problems
			Biotransformation of PPIs
			Recommendations for practice
			Start a conversation and detect problems
4	Application of low molecular weight heparins	Application skills	Prevalence and indications of heparins
			Common problems
			Recommendations for practice
			Start a conversation and detect problems
5	Interaction between low dosed acetylsalicylic acid (ASA) and ibuprofen	Time of application, drug-drug-interaction	Prevalence of ASA and ibuprofen
			Explanation of interaction’s mechanism and impact
			Safety aspects of nonsteroidal anti-inflammatory drug(s) and factors influencing them
			Recommendations for practice
			Start a conversation and detect problems
6	Special considerations when taking levodopa	Time of application, drug-food-interactions	Prescription prevalence and indications
			Common problems
			Recommendations for practice
			Start a conversation and detect problems
7	Swallowing problems with tablets	Application skills	Principles of swallowing
			Causes of swallowing problems
			Recommendations for practice
			Start a conversation and detect problems
8	Recognize and resolve constipation as an adverse drug reaction	Avoidance of adverse drug events	Definition of constipation
			Casualty assessment of adverse drug events leading to constipation
			Recommendations for practice
			Start a conversation and detect problems
9	Resorption of direct oral anticoagulants (DOACs)	Drug-food-interactions, application skills	Prevalence and indications of DOACs
			Pharmacodynamic and pharmacokinetic aspects of individuals DOACs
			Factors that influence DOACs’ resorption
			Start a conversation and detect problems
10	Special considerations when taking bisphosphonates	Time of application, drug-food-interactions	Prevalence and indications of bisphosphonates
			Common problems
			Recommendations for practice
			Start a conversation and detect problems

**Fig. 1:**
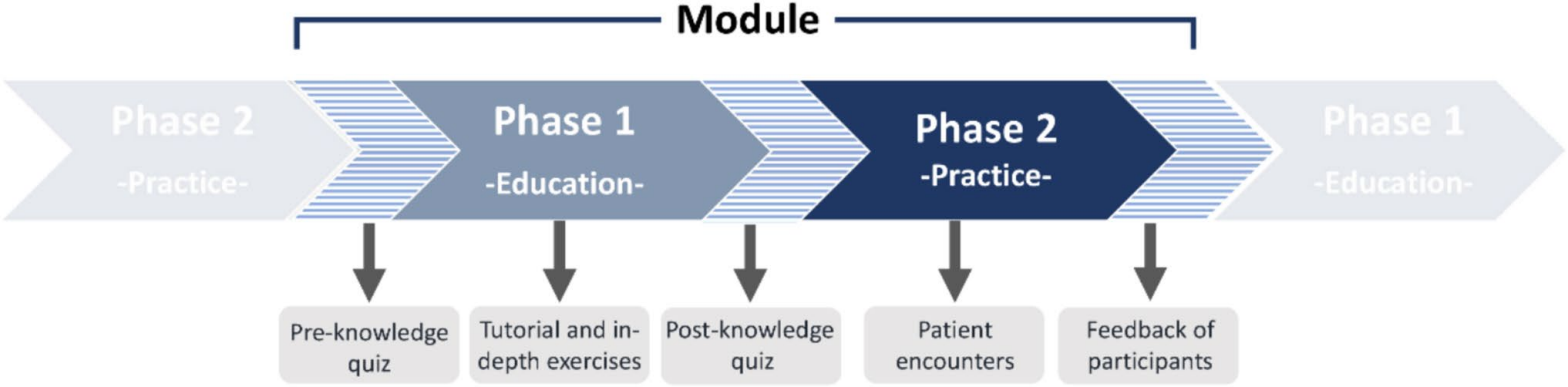
Structure of the e learning program. Each module is structured in an educational (quizzes, tutorials, in-depth exercises or patient brochures) and a practical phase (documentation of anonymized patient encounters and feedback from participants)

All modules followed the same step-by-step procedure (Fig. 1), and after each step, study personnel sent invitation links via mail to the next step. The educational phase (Phase 1) started with a pre-knowledge quiz to determine what the participants already knew about the DRP. Then, participants got access to a 10–15-min tutorial (audio-visual PowerPoint presentation (Microsoft Office Professional 2016, Redmond, Washington, USA)). Optional in-depth exercises for self-study were provided. In some modules, patient brochures were offered. The educational phase ended with the post-knowledge quiz asking questions similar to context and difficulty of the pre-knowledge quiz to assure comparability. The participants could contact study personal in case of questions.

In the practical phase (Phase 2) participants were asked to document at least 5 anonymous patient encounters related to the DRP. For this purpose, documentation forms were designed that included at least:

- Identification number of the drug(s) involved,
- Whether DRPs were identified in the patient encounters,
- A brief description of the intervention(s) taken to resolve the DRP(s),
- And whether the DRPs were resolved in the participant's opinion.

Each documentation form contained brief instructions for completion; participants did not receive any additional training. Submission of the documentation form activated the next module. At the end of Phase 2, participants could provide anonymous feedback on the module and how applicable they found the recommendations for practice (5-point-Likert scale: fully agree, rather agree, partly, rather not agree, not agree). Frequency of predetermined reasons for not resolving DRPs was asked (5-point-Likert scale: never, rarely, sometimes, often or always happened). The reasons were: lack of understanding on the part of the patient (in terms of language or content); necessary consultation with the physician was not possible; the problem was too complex for the pharmacy; consultation with the physician did not result in a change of prescription or patient's behavior; and uncertainty among CPs regarding how to solve a problem. There was also an option to add additional reasons under ‘Other’. Suggestions for improvement were collected using free text or multiple-choice answers.

Module 1 was pre-tested with 3 CPs with regard to the clarity and quality of the content, including the exercises. Their feedback was also used for the development of the future modules which followed a predefined checklist including several internal checks and feedback rounds involving several members of the study team also including colleagues working in community pharmacies to support the practical relevance of the content. In addition, the modules were developed consecutively, allowing feedback from the previous modules to be directly integrated in subsequent ones (e.g., the pre-knowledge quiz was reduced from 7 questions in Module 1 to 5 questions in Module 2).

Having completed all modules, participants took a final quiz to assess how much of the new knowledge they had retained. It included 2 single-choice questions per module (i.e., 20 questions in total).

### Data collection

Answers of the knowledge quizzes and feedback responses were collected via the online survey platform SurveyMonkey (SurveyMonkey Europe UC, 2017-2018 Dublin, Ireland) and automatically transferred to Excel sheets (Microsoft Office Professional 2016, Redmond, Washington, USA). Participants were asked to send documentation forms via mail or fax. They were manually transferred to Excel sheets.

### Data analysis

All questions on a quiz had to be completed in order to be included in the analysis. When participants completed the same quiz multiple times, only the first completed entry was considered. Regardless of whether previous Modules had been completed, full answers to subsequent quizzes were included. For each quiz, a score was calculated representing the relative frequency of correctly answered questions to the total number of questions in the quiz. Comparisons between pre- and post- knowledge scores were calculated using the Wilcoxon test. All p-values were interpreted exploratively, with *p* < 0.05 set as statistically significant.

From the documentation forms, entries with conflicting answers (e.g., ‘yes’ and ‘no’ selected for whether a problem was present) were excluded from analysis. Documented encounters were classified according to the German version 9.1 of the Pharmaceutical Care Network Europe (PCNE) system [4]. Based on the description of the DRP or intervention, it was evaluated if a DRP was ‘manifest’ or ‘potential’. In some cases, the assignment of PCNE codes was ambiguous or allowed for multiple interpretations, thus, specific coding rules were established that specified which code to use when it was unclear which code should be assigned in a particular case and to distinguish certain codes from one another (Supplement B). Classified DRPs and participants’ feedback were analyzed descriptively. Missing values were not imputed; instead, the absolute number of available entries was reported, and subsequent analyses were calculated on these absolute numbers.

IBM SPSS Statistics Version 28.0 (IBM SPSS Statistics, Armonk, New York, USA, 2021) and Microsoft Excel 2019 (Microsoft Office Professional 2016, Redmond, Washington, USA) were used. Plotting of figures was also done with R studio Version 4.3.1 using the packages *tidyverse* and *ggplot* [27].

### Ethics approval

This study was performed in line with the principles of the Declaration of Helsinki. Ethical approval was obtained from the Medical Faculty of the University Hospital Heidelberg (approval reference number S-607/2016).

## Results

### Participants

The chamber of pharmacists Baden-Wurttemberg invited about 10,000 CPs. Of those, 203 consented. About two-thirds of participants were employed (65.5%, n = 133/203), followed by pharmacy owners (18.7%, n = 38/203) and branch managers (15.8%, n = 32/203). The number of participants declined over the course of the modules. In Module 1, 184 participants answered at least the pre-knowledge quiz while only 119 participants did so in Module 10 (Supplement C). The final quiz was completed by 70 participants. 48 participants completed all quizzes.

### Comparison of knowledge gain with quizzes (Kirkpatrick’s level ‘knowledge and skill acquisition’)

**Fig. 2:**
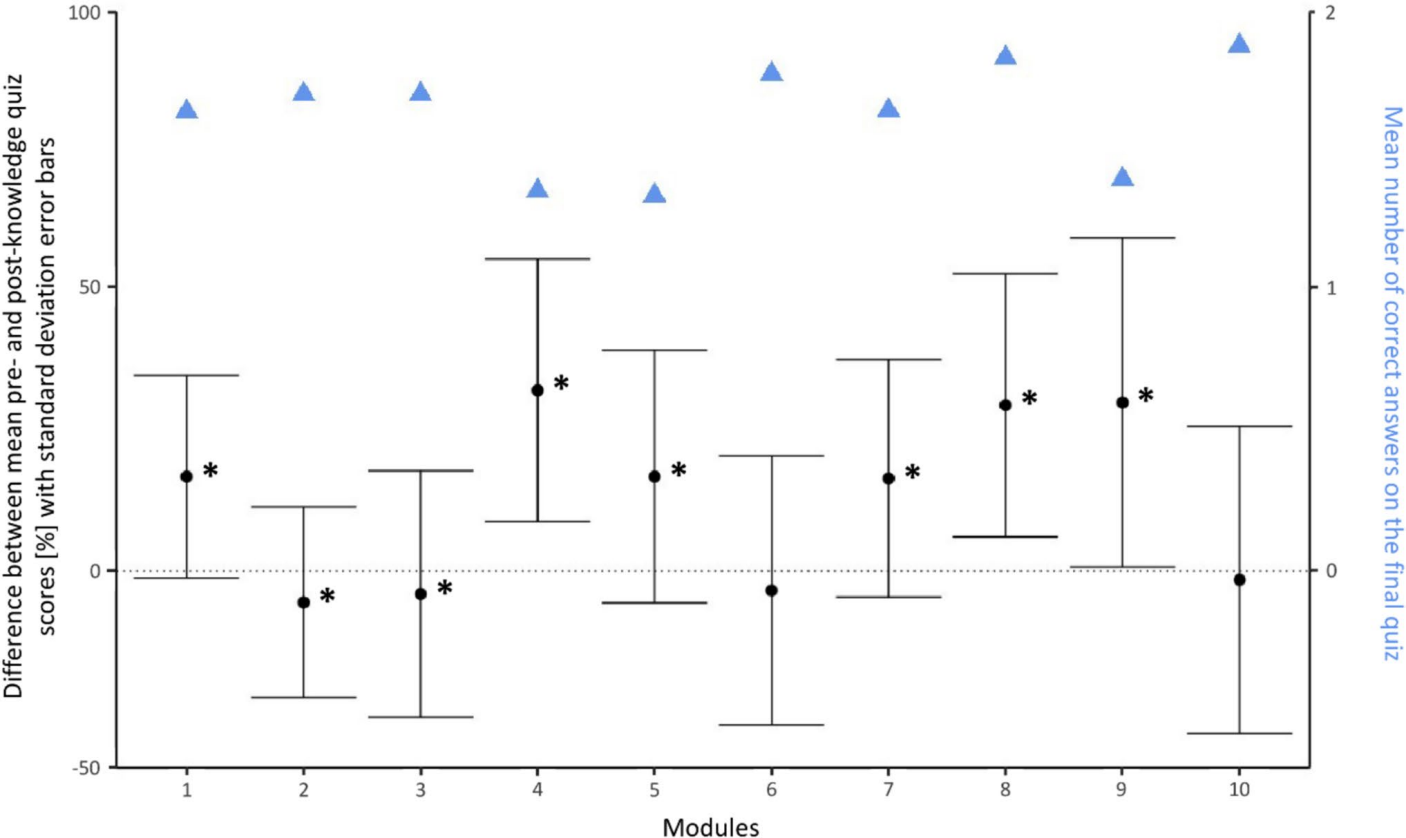
Difference between pre-knowledge and post-knowledge quiz mean scores [%] (N = 1231) with standard deviation bars and mean number of answers that were given correctly in the final quiz (N = 70) for each of the 10 e learning modules (triangles). The mean score was derived by comparing the proportion of correct answers in the tests to the total number of questions. *Significant change comparing pre- and post-knowledge quiz mean scores with Wilcoxon test (*p*-values < 0.05 were considered statistically significant).

A total of 1231 entries were available for the analysis of participants’ knowledge change; that is, participants who completed both the pre- and post-knowledge quizzes. The mean score for the pre-knowledge quiz across all modules was 74.8% ± 23.5%, and for the post-knowledge quiz 87.2% ± 17.1% (*p* < 0.001; details for each module are presented in Supplement D). The highest increase in pre- vs. post-knowledge by mean score was 32.3% ± 23.2% (*p* < 0.001) in Module 4, followed by 29.4% ± 29.1% (*p* < 0.001) in Module 9, and 29.3% ± 23.2% (p < 0.001) in Module 8 (Fig. 2). In about half of the entries (50.1%, n = 617/1231), participants improved their knowledge from pre- to post-knowledge quizzes, with most of the participants improving in the same modules that showed the largest overall knowledge increase (i.e., Module 4 (83.5%, n = 96/115); Module 8 (80.2%, n = 97/121); Module 9 (69.6%, n = 80/115)). On average, the 70 participants answered 1.63 ± 0.61 out of 2 correctly in each module in the final quiz.

### Documentation of the identified problems and interventions taken (Kirkpatrick’s level ‘workplace application’)

In total, 13,778 patient encounters were documented, most being reported in Module 2 (n = 2320), and least in Module 10 (n = 715). The median number of encounters reported by a participant across all modules was 71 (interquartile range: 77.8), with a maximum of 299. According to the participants, in 37.5% (n = 5173/13,778) of all encounters at least one DRP was identified, regardless of whether the DRP was anticipated in the individual module, and in 7.9% (n = 1102/13,778) it was unknown whether a problem existed. Across all modules, participants reported the most problems in Module 7 (62.8%, n = 622/991) and Module 5 (53.4%, n = 622/1164).

**Fig. 3:**
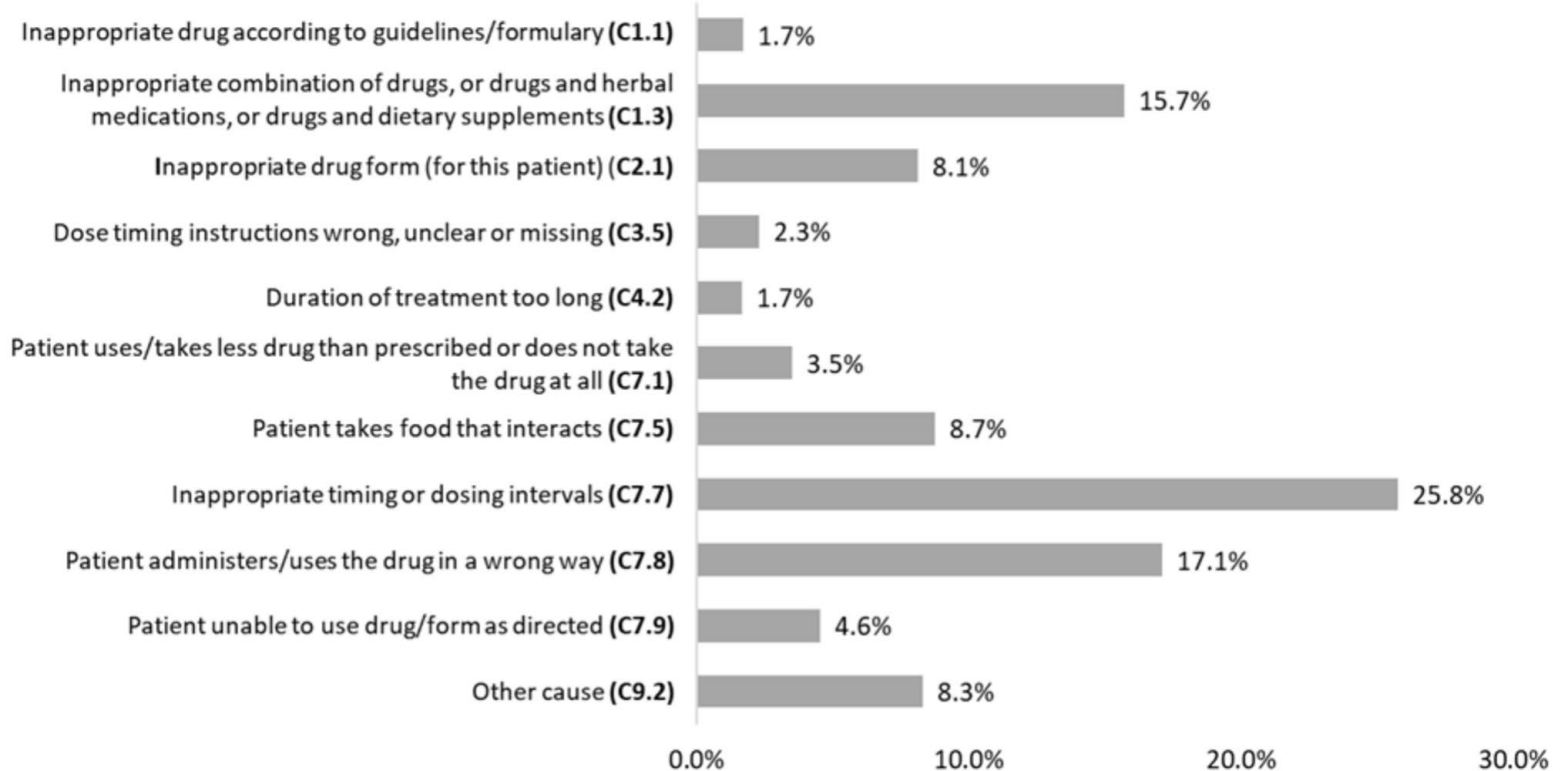
Most frequent causes of drug-related problems (DRPs; N = 10,087). Only causes that occurred more than 1% are represented.

According to the PCNE classification codes applied to the patient encounters, a total of 6679 DRPs were identified, with an average of 1.1 ± 0.3 per patient encounter. The most common *problem* was ‘effect of drug treatment not optimal (P1.2)’ (70.1%, n = 4679/6679), followed by ‘adverse drug event (possibly) occurring (P2.1)’ (28.9%, n = 1927/6679). In 18.9% (n = 1262/6679) the DRP was ‘manifest’. The most frequent *causes* across all modules are presented in Fig. 3. The frequency of causes for each module is shown in Supplement E (Figure E1). The most common *intervention* was ‘patient (drug) counselling (I2.1)’ (64.9%, n = 5432/8375; Figure E2 in Supplement E).

In 7688 patient encounters, participants indicated whether or not the DRP had been resolved or if its resolution status was unknown. However, in 2615 of those encounters, participants reported that a problem was solved, not solved or it was unknown although they initially did not report a problem, leaving 5073 encounters with actual documented problem. Of those, participants indicated in 89.6% (n = 4550/5073) that problems could be solved. In 3.1% (n = 155/5073) of these encounters, the problem was not solved according to the participants, and in 7.3% (n = 368/5073) the resolution status was unknown. Resolution rates were especially high in Module 2 with 94.1% (n = 668/710), in Module 6 with 89.2% (n = 223/250), in Module 10 with 89.5% (n = 188/210). In comparison with participants’ self-assessment of whether the DRP was resolved, the PCNE-coded status indicated that the DRP was 'totally solved' (O1.1) in 86.4% (n = 5773/6679), and the most common acceptance rate was ‘intervention accepted, implementation unknown (A1.4)’ with 84.0% (n = 5612/6679).

### Participants’ feedback on the e-learning program (Kirkpatrick’s level ‘participant satisfaction’)

A total of 1128 feedback responses were received. The majority of participants ‘rather’ or ‘fully agreed’ that the program has helped them to approach patients about possible DRPs (84.8%, n = 956/1127), and to identify patients, situations or prone drugs belonging to DRPs (80.6%, n = 907/1126). A total of 68.0% (n = 765/1125) of participants agreed that the conversation starters were helpful in patient encounters. Most participants (80.9%, n = 908/1122) reported that they could easily implement the provided recommendations in their practice.

According to the participants, the most common reasons for unsolved DRPs, that in 17.3% (n = 194/1120) ‘sometimes’, in 5.0% (n = 56/1120), or in 0.6% ‘always happened’, was a lack of understanding on the part of the patient (language or content wise). Followed by that a necessary consultation with the physician was not possible (of ‘sometimes’: 5.4%, n = 109; ‘often’ 2,3%, n = 25; ‘always’ 1.0%, n = 11 of 1102 responses) and the problem was too complex for the pharmacy setting (‘sometimes’: 6.5%, n = 72; ‘often’ 1.9%, n = 21; ‘always’ 1.2%, n = 13 of 1112 responses). However, the predetermined reasons against solving DRPs were mostly ‘rare’ or ‘non-existent’ (88.7%, n = 4900/5523).

As a result of the e-learning, participants ‘rather’ or ‘fully agreed’ that they felt more confident in identifying and resolving DRPs (96.0%, n = 1078/1123; 5 missing answers), and also in their general counseling skills (87.0%, n = 981/1127; 1 missing answer). They wanted to implement the training in their future practice (‘rather’ or ‘fully agreed’ in 97.9%, n = 1101/1125; 3 answers missing). Almost all participants (‘rather’ or ‘fully agreed’ in 94.8%, n = 1069/1128) would recommend the e-learning to their colleagues. To improve the modules, participants suggested making more patient brochures (23.6%, n = 266/1128), and integrate more in-depth exercises (16.1%, n = 182/1128). Yet, 62.2% of the participants (n = 702/1128) said that they liked the modules just the way they were.

## Discussion

This study evaluated the development and the overall pilot-testing of an e-learning program designed to improve CPs’ knowledge of DRPs and, ultimately, their ability to manage them. Improvements were observed on all 3 assessed levels of the Kirkpatrick model.

At level 1, participant feedback was predominantly positive, which is in line with previous e-learnings [28–31]. Particularly, blended learning approaches, combining e-learning with practical assessments such as Objective Structured Clinical Examinations (OSCEs), were well received and considered successful for skill development [29]. These approaches, i.e. the link of teaching material with actual practical application, may also have been successful in this study.

At level 2, the significant improvement in the pre- and post-knowledge quizzes suggests that the program effectively conveyed learning objectives, consistent with other examples in the literature. A systematic review reported in 8 of 9 studies improvements in participants’ knowledge [32]. Of note, in our study, a slight decrease in knowledge was observed in Modules 2 and 3. However, this may not be related to the content of the Modules themselves, but rather to the quiz questions. For example, many participants answered one particular post-knowledge quiz question in Module 2 incorrectly, suggesting that it was particularly challenging. Additionally, in this study, a relatively low knowledge increase was observed in some modules—particularly in those where baseline levels were already high, pointing to a ceiling effect. Due to high baseline knowledge, and a potential an insufficiently sensitivity of the quizzes, it is assumed that there was limited measurable improvement. To address this, the program could benefit from first assessing participants’ prior knowledge and then adapting the quizzes accordingly. The high scores in our final quiz suggest that participants retained the knowledge acquired during the program. Lalonde and colleagues also observed an improvement in knowledge and clinical skills after 12 months of participating in a similar continuing education program [18]. However, while knowledge seems to be robust, improvements in practical skills and real-world dispensing behaviors are less consistently studied or observed [22, 33], indicating a need for additional educational strategies to translate knowledge into practice.

Level 3 was evaluated through the documentation of patient encounters. The substantial number of documented encounters indicates that this task was feasible. This also reinforces the decision to select DRPs that could be effectively addressed in the pharmacy. At the same time, CPs also identified other, perhaps more complex DRPs which needed involvement of physicians what had been originally not anticipated. However, the majority of reported DRPs were successfully resolved. Kimberlin et al. also found that pharmacists who participated in an educational intervention program were more likely to detect and manage DRPs. This difference persisted during a 3-month follow-up [34]. As this e-learning required CPs to apply their knowledge, it could be argued that knowledge acquisition was more effective [35, 36]. Supporting this, a systematic review identified reinforcement of learning, through assessments, feedback, and peer exchange, as important for pharmacists engaged in professional development [17]. Moreover, it may be assumed that participants with greater knowledge improvement were also more likely to detect and resolve DRPs in practice. Williams and colleagues reported as part of a continuing education study a moderate but statistically significant correlation between pharmacists’ knowledge scores, and their clinical intervention rates. In their study, clinical interventions were defined as the identification of an actual or potential DRP followed by pharmacist recommendations to resolve or prevent it [37].

In our study, it was observed that DRPs relating to the patient as source were more easily to detect and address, since they can be solved in a singular intervention within a pharmacy consultation. On the other hand, DRPs caused by failures on the level of ‘prescribing and drug selection’ (C1) often require more complex clinical judgment. This has been also observed in other studies [38, 39].

Although direct patient health outcomes were not captured, which would have been the level 4 of Kirkpatrick's evaluation model, a substantial proportion of reported DRPs could be resolved according to the participants. In the literature, it has been demonstrated that CP-led interventions aimed at identifying and resolving DRPs, such as medication reviews, can positively influence intermediate outcomes like biomarkers or surrogate parameters [10]. Thus, it may be postulated that the impact of the program on patient safety was positive in some degree. However, this of course, needs to be proven in a future evaluation.

### Future implications of the e-learning program

Since 2019, the Chamber of Pharmacists Baden-Wurttemberg has offered the program in cooperation with the Cooperation Unit Clinical Pharmacy Heidelberg as a regular continuing training measure to all their CP members. The modules are continuously updated and expanded, including the transfer of the program into a homepage format, which facilitates organisation.

### Strengths and limitations

The first limitation is that the knowledge quizzes were not validated; thus, it is unclear how meaningful the learning progress really was. Although efforts were made to balance the number of simpler and more complex questions within each module, the comparability of the quizzes remains questionable. Secondly, the participation was voluntary, likely attracting highly motivated CPs, potentially with above-average baseline knowledge facilitating ceiling effects. Third, the documentation of patient encounters was not enforced to be consecutive, i.e., participants could choose which encounters to document. While this approach captured a range of situations (no problem, problem resolved, and problem not resolved) resolution rates cannot be generalized. Additionally, because participants did not receive formal training on how to complete the forms, the data is subject to a range of additional external influences. Finally, the documentation forms used in this study were not exclusively designed to be later on assessed using the PCNE classification, which may have led to the fact that the documented data were not always perfectly suited for the classification.

The study has also several strengths. Firstly, more participants than initially targeted were enrolled, reflecting the interest in and relevance of the e-learning. Additionally, the majority of dropouts occurred after Module 1, suggesting that these participants discontinued the program because it did not meet their initial expectations. In contrast, participants who progressed completed most often all modules, indicating that the program is feasible. Secondly, data were collected at multiple Kirkpatrick evaluation levels, suggesting the success of the educational program at all these levels. To strengthen the evidence further, subsequent studies should consider including a control group and explore objective measures of clinical impact.

## Conclusion

The developed e-learning program is a well-received approach to further enhancing CPs’ knowledge about DRPs and how to solve them. The participants also accepted the special aspect of documenting patient encounters well, which may have fostered a future transfer of knowledge. Integrating such a program into continuing professional development initiatives may contribute to optimized pharmaceutical care.

## Supplementary Information

Below is the link to the electronic supplementary material.

Supplementary file1 (PDF 498 KB)

## Data Availability

Ethics approval has not foreseen sharing of data. However, in case of a reasonable request with regard to data integrity, data will obviously be made available.
