# Supplementary material for: Development and evaluation of an e-learning program on drug-related problems for community pharmacists
Source: Int J Clin Pharm. 2026 Mar 25;48(4):1349–58. doi: 10.1007/s11096-026-02111-5 (PMC13369537; doi:10.1007/s11096-026-02111-5)
Supplement: Supplementary file 1 — Supplementary file1 (PDF 498 KB) [file 11096_2026_2111_MOESM1_ESM.pdf]

Development and evaluation of an e-learning program on drug-related problems for community pharmacists

Cathrin J. Vogt[1]*, Alesia Reuther[1]*, Viktoria S. Wurmbach[1], Marina Weißenborn[1], Janina A. Bittmann[1], Katharina Wien[1], Anette Lampert[1], Emilia Maria Boček Eknes[1,2], Patrick Schäfer[3], Hanna M. Seidling[1]**

[1]Heidelberg University, Medical Faculty Heidelberg / Heidelberg University Hospital, Internal Medicine IX - Department of Clinical Pharmacology and Pharmacoepidemiology, Cooperation Unit Clinical Pharmacy, Germany

[2]Centre for Pharmacy, University Bergen, Bergen, Norway

[3]Chamber of Pharmacists Baden-Wurttemberg, Stuttgart, Germany

*Equally contributed

**Corresponding Author / Contact information:

Hanna M. Seidling

E-Mail: Hanna.seidling@med.uni-heidelberg.de

Postal address: Heidelberg University, Medical Faculty Heidelberg / Heidelberg University Hospital, Internal Medicine IX - Department of Clinical Pharmacology and Pharmacoepidemiology, Cooperation Unit Clinical Pharmacy, Im Neuenheimer Feld 410, Heidelberg 69120, Germany

Supplementary material

A. DoCTRINE Reporting guideline

B. Coding rules

C. Overview of participation in the individual tasks of the modules

D. Comparison between pre- and post-knowledge test scores by module

E. Prevalence of identified causes of drug-related problems and interventions taken, as classified by the PCNE system

Supplement A: Defined Criteria To Report INnovations in Education (DoCTRINE)

Citation: Blanco M, Prunuske J, DiCorcia M, Learman LA, Mutcheson B, Huang GC. The DoCTRINE Guidelines: Defined Criteria To Report INnovations in Education. Academic Medicine. 2022 May 1;97(5):689-695.

**Introduction**	**Y/N**
1. Need for the curriculum	Y
2. Review of relevant literature, theories, models, or published curricula	Y
3. Unique contribution of the curriculum to the literature	Y
**Curriculum Development**	**Y/N**
4. Purpose/goals of the curriculum	Y
5. Outcome-based learning objectives	Y
6. Target population of learners	Y
**Curriculum implementation**	**Y/N**
7. Instructional setting for curriculum delivery	Y
8. Resources for implementing the curriculum	Y
9. Description of instructional methods	Y
10. Methods to evaluate achievement of outcome-based learning objectives	Y
11. Origin of evaluation instrument(s)	N/A
**Results**	**Y/N**
12. Number of learners participating in the curriculum	Y
13. Number of participants included in the evaluation	Y
14. Evidence of achievement of outcome-based learning objectives	Y
**Discussion**	**Y/N**
15. Summary of findings	Y
16. Interpretation of findings in relation to the existing literature	Y
17. Lessons learned from the implementation of the curriculum	Y
18. Limitations of the evaluation of the curriculum	Y
19. Describes future implications of the curriculum	Y

Supplement B: Coding Rules. ***Presented are quotes from the documentation forms or descriptions from the research team indicating the situations in which a specific PCNE code was applied.***

	Description	General	Module 1	Module 2	Module 3	Module 4	Module 5	Module 6	Module 7	Module 8	Module 9	Module 10
**General**										Lifestyle problems not classified as DRP		
**Unknown**	Not possible to apply classification because of missing information											
**P1.1**	No effect of drug treatment despite correct use	"Therapy not well adjusted"										
**P1.2**	Effect of drug treatment not optimal	Food-drug-interactions; wrong timing; patient does not take medication as prescribe; unauthorized administration via feeding tube	Too short interval between different eye drops (dilution effect) [together with P2.1]; still dry eyes; correct application was generally unknown [together with P 2.1];	Combination of polyvalent cations with complex builders like L-Thyroxine		Correct application was generally unknown [together with P 2.1]; Removal of air bubble in syringe	Interaction between ibuprofen and acetylsalicylic acid	On-off-phenomenons	Patient does not take medication			

	Description	General	Module 1	Module 2	Module 3	Module 4	Module 5	Module 6	Module 7	Module 8	Module 9	Module 10
			bitter taste in mouth after application [together with P 2.1]									
**P1.3**	Untreated symptoms or indication	Only if not already recognized as a symptom by the doctor				Fear of syringes [together with P2.1]						
**P2.1**	Adverse drug event (possibly) occurring		(Potential drug-drug-interaction) ; nasolacrimal occultation not performed; Lack of hygiene; bitter taste in mouth after application [together with P 1.2]			Correct application was generally unknown [together with P 1.2]; patient feels unsafe during application	Ibuprofen in chronic use	On-off-phenomenon		Constipation with causality check (manifest problem), if no causality check, problem as potential		
**P3.1**	Unnecessary drug-treatment		Too many eye drops during one application									

	Description	General	Module 1	Module 2	Module 3	Module 4	Module 5	Module 6	Module 7	Module 8	Module 9	Module 10
**P3.2**	Unclear problem/complaint	Problems appeared to a child										
**C1.1**	Inappropriate drug according to guidelines/formulary				Ibuprofen and metamizole in chronic use							
**C1.3**	Inappropriate combination of drugs, or drugs and herbal medications, or drugs and dietary supplements			Combination with of polyvalent cations and L-Thyroxine [together with C7.7]	Combination of L-Thyroxine and iron [together with C7.7]		On-demand medication [together with C7.7]					
**C1.4**	Pseudo-) Doppelmedikation	Together with P2.1										
**C2.1**	Inappropriate drug form/formulation (for this patient)										Problems with swallowing medication	
**C3.1**	Drug dose too low							“Therapy not well adjusted”				
**C3.2**	Drug dose of a single active ingredient too high									Constipation due to increased drug dose		
**C3.5**	Drug dose of a single active ingredient too high	Problem is on prescriber level &	information about the distance between		The patient was unaware of the correct	Prescription of emergency ambulance	Patient applies medication correctly,					

	Description	General	Module 1	Module 2	Module 3	Module 4	Module 5	Module 6	Module 7	Module 8	Module 9	Module 10
		patient was not correctly educated by first prescription	individual eye drops was too short		application and dosage.		but according to the wrong medication plan					
**C4.2**	Duration of treatment too long						Ibuprofen in chronic use					
**C5.2**	Necessary information not provided or incorrect advice provided	Problem is on pharmacist level	Dosage unclear									
**C7.1**	Patient intentionally uses/takes less drug than prescribed or does not take the drug at all for whatever reason	Forgotten to take the medication	Treatment duration to short	Irregular use of medication [together with C7.7]	Use of medication only when required [together with C7.7]				if explicitly described that patient does not/irregularly use medication	Cause due to eating habits		
**C7.5**	Patient takes food that interacts	Intake with food / not enough time apart from food		Intake together with coffee with milk [together with C7.7]	Intake with/after a meal			Intake with/after a meal			Clarification about intake with food	
**C7.6**	Patient stores drug inappropriatel		Durability, storage in refrigerator [together with C2.1]									

	Description	General	Module 1	Module 2	Module 3	Module 4	Module 5	Module 6	Module 7	Module 8	Module 9	Module 10
**C7.7**	Inappropriate timing or dosing intervals		Insufficient distance between individual eye drops [together with C7.8]; patient does not use medication regularly	Distance to L-Thyroxine intake to short; irregular use [together with C7.1]; intake together with coffee with milk [together with C7.5]	Intake at the wrong time; advice to take on an empty stomach during interventio n; intake of iron and L-thyroxine [together with C1.3]		Ibuprofen and ASS taken together; on-demand medication [together with C1.3]					
**C7.8**	Patient unintentionall y administers/us es the drug in a wrong way	Only if the application is incorrect: e.g., injection syringe used incorrectly	Eye closure or nasolacrim al occlusion not performed						Patient crushed medication; drank too little water after medication intake			Patient crushed medication; drank too little water after medication intake
**C7.9**	Patient physically unable to use drug/form as directed		Not for children (see C9.2); patient cannot open the screw cap						Only if explicitly described that the patient is too old / has e.g., gag reflex., otherwise only C2.1			Difficulty in swallowing could indicate that patient had an oesophagea l irritation [together with C2.1]

	Description	General	Module 1	Module 2	Module 3	Module 4	Module 5	Module 6	Module 7	Module 8	Module 9	Module 10
**C7.10**	Patient unable to understand instructions properly		Not for children (see C9.2);									
**C9.1**	No or inappropriate outcome monitoring (incl. TDM)											
**C9.2**	Other cause; specify	Problems appeared by children								Adverse drug reaction		
**I0.1**	No Intervention	Only if it states that no interventio n was carried out		Patients states "Use should remain as it has been for years"								
**I3.3**	Formulation changed to …		"Switch from single dose to multiple dosage forms"									
**I3.4**	Instructions for use changed to …	Only if instructions were also correct beforehand, not if patient applied medciation incorrectly and was then										

	Description	General	Module 1	Module 2	Module 3	Module 4	Module 5	Module 6	Module 7	Module 8	Module 9	Module 10
		corrected, in this case = I2.1										
**I3.6**	Drug started								Medcoat® to help with swallowing solid medication			
**A1.4**	Intervention accepted, implementation unknown	Problem was solved and the intervention described, but no further explanation if patient adhered to recommendation										
**A2.4**	Intervention not accepted: unknown reason	Together with O3										
**A3.1**	Intervention proposed, acceptance unknown	Together with O0.1										
**A3.2**	Intervention not proposed											
**O0.1**	Problem status unknown	Acceptance is not mentioned in the description										

	Description	General	Module 1	Module 2	Module 3	Module 4	Module 5	Module 6	Module 7	Module 8	Module 9	Module 10
		of the intervention										
**O3.4**	No need or possibility to solve problem	Together with I0.1 and A3.2										

Supplement C. Overview of participation in the individual tasks of the modules

	Number of participants (n) that completed the pre-knowledge quiz (N=1,387)	Number of participants (n) that completed the post-knowledge quiz (N=1,231)	Number of documented patient encounters (n) (N=13,761)	Number of participants (n) that documented patient encounters (N=1,255)	Number of participants (n) that completed the feedback (N=1,128)
Modul 1	184	154	2243	160	150
Modul 2	157	138	2303	141	121
Modul 3	139	122	2281	137	124
Modul 4	138	115	1019	130	111
Modul 5	137	127	1164	134	110
Modul 6	132	116	762	111	110
Modul 7	130	116	991	117	106
Modul 8	128	121	1138	113	102
Modul 9	123	115	1145	114	94
Modul 10	119	107	715	98	100

Supplement D. Comparison between pre- and post-knowledge test scores by module

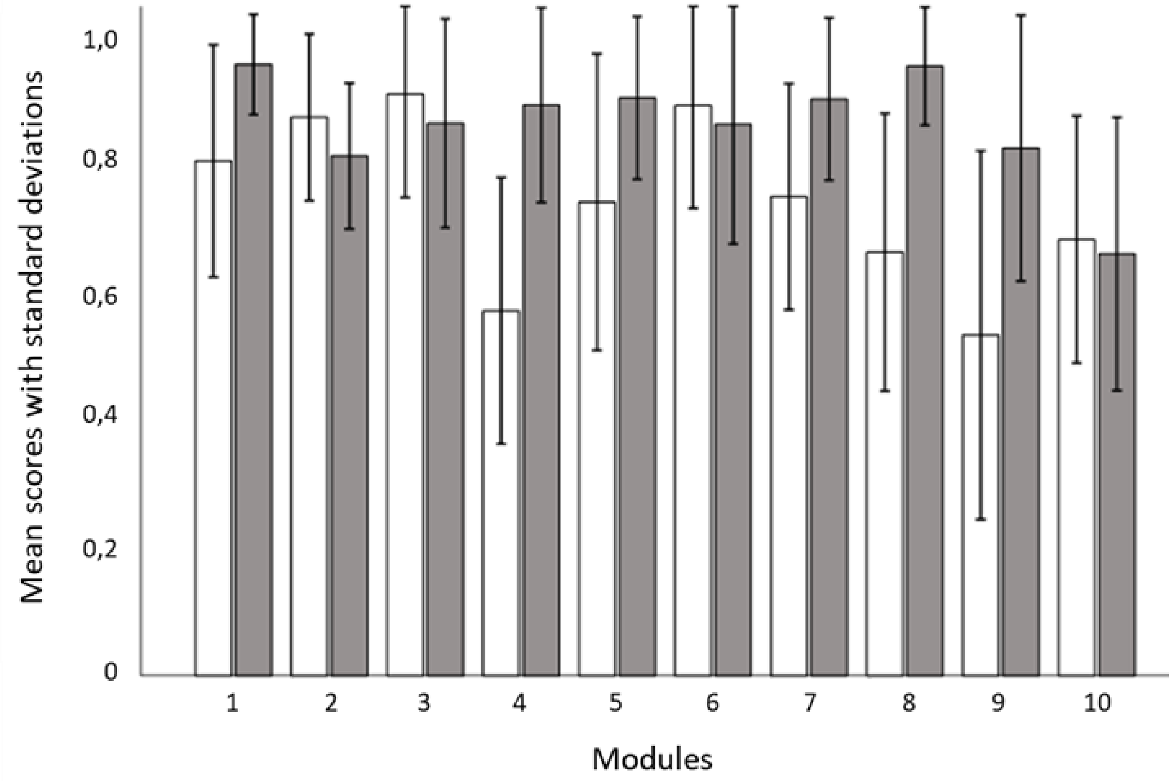

Figure D1: Comparison of the mean pre- and post-knowledge test scores with standard deviations by each module of the eLearning.

Supplement E. Prevalence of identified causes of drug-related problems and interventions taken, as classified by the PCNE system

		Module									
		1	2	3	4	5	6	7	8	9	10
Total number of causes per module		*1549*	*1898*	*1036*	*594*	*1937*	*625*	*1467*	*618*	*208*	*155*
**Causes of DRPs coded by PCNE classification Version 9.0**		Percentages [%]									
C1.1	Inappropriate drug according to guidelines/formulary	0.1				8.7			0.2	1.0	
C1.2	No indication for drug		0.1	0.2	0.2	0.1				1.0	
C1.3	**Innapropriate combination of drugs, or drugs and herbal medications, or drugs and dietary supplements**	0.1	33.0	2.2		41.4	11.0			2.4	**39.4**
C1.4	Inappropriate duplication of therapeutic group or active ingredient	0.1		0.1		0.1				1.4	
C1.5	No or incomplete drug treatment in spite of existing indication	0.1		0.1							
C2.1	**Inappropriate drug form (for this patient)**	2.3		0.3	0.5	0.1		51.3		9.1	4.5
C3.1	Drug dose too low			0.2			0.8				
C3.2	Drug dose of a single active ingredient too high									1.0	
C3.3	Dosage regimen not frequent enough						1.0				
C3.4	Dosage regimen too frequent	0.1		0.2							
C3.5	Dose timing instructions wrong, unclear or missing	1.0	0.3	11.6	10.4	0.2	0.5			7.7	1.9
C4.1	Duration of treatment too short			0.1							
C4.2	Duration of treatment too long	0.2		0.4	0.2	8.1			0.3	0.5	
C5.1	Prescribed drug not available	0.1			0.2						
C5.2	Necessary information not provided or incorrect advice provided	0.3			0.2					12.5	4.5
C5.4	Wrong drug or strength dispensed				0.2						
C6.1	Inappropriate timing of administration or dosing intervals			0.1			1.8				
C6.6	Drug administered via wrong route	0.1								0.5	
C7.1	**Patient intentionally uses/takes less drug than prescribed or does not take the drug at all for whatever reason**	0.3	0.1	14.5	5.4		0.3	10.8			2.6
C7.2	Patient uses/takes more drug than prescribed	1.5		0.1							
C7.3	Patient abuses drug (unregulated overuse)	0.1							0.2		
C7.4	Patient uses unnecessary drug	0.1									
C7.5	**Patient takes food that interacts**		16.6	20.1			38.1			39.9	23.9
C7.6	Patient stores drug inappropriately	4.5			0.3						
C7.7	**Inappropriate timing or dosing intervals**	4.5	49.1	48.1	0.5	41.3	45.1			5.8	1.9
C7.8	**Patient unintentionally administers/uses the drug in a wrong way**	75.7	0.1	0.6	72.2			4.2	0.0	14.9	16.1
C7.9	Patient physically unable to use drug/form as directed	3.6			1.7		0.2	26.6		0.5	1.3
C7.10	Patient unable to understand instructions properly		0.1		0.5		0.3	0.8			
C9.1	No or inappropriate outcome monitoring (incl. TDM)					0.1				0.5	
C9.2	**Other cause**	5.2	0.1	0.4	7.1	0.2		6.4	99.4		
C9.3	No obvious cause	0.2		0.2	0.2		0.2			0.5	2.6
**Non-codable cause**			0.5	0.7	0.3		0.8			1.0	1.3

Figure E1: Prevalence (in percentages) of identified causes of drug-related problems in each e-learning module. The darker the color, the more frequently the cause occurred in the respective module. Only causes with at least one identified case are displayed, as well as percentages >0.0%.

		Module									
		**1**	**2**	**3**	**4**	**5**	**6**	**7**	**8**	**9**	**10**
Total numbers of interventions per module		***1555***	***996***	***844***	***648***	***1028***	***308***	***967***	***789***	***213***	***147***
**Interventions coded by PCNE classification Version 9.0**		Percentages [%]									
**I0.1**	No Intervention	0.1	1.0	0.5		1.1		0.2	6.7	0.5	
**I1.1**	Prescriber informed only			0.1		0.3		0.1			
**I1.2**	Prescriber asked for information	0.1					0.3				
**I1.3**	Intervention proposed to prescriber					0.8			0.6		
**I1.4**	Intervention discussed with prescriber	0.3	0.5	1.1	0.9	4.4	1.0	8.7	2.5	4.7	
**I2.1**	**Patient (drug) counselling**	81.4	90.5	85.1	68.2	79.3	88.0	37.2	32.3	83.1	93.2
**I2.2**	**Written information provided (only)**	10.7	2.6	0.2	25.0	0.2	3.2	1.8		0.5	
**I2.3**	Patient referred to prescriber	0.6	0.3	1.9	0.3	2.2	2.6	0.6	3.2	4.2	1.4
**I2.4**	Spoken to family member/caregiver	0.3	0.2	0.7	2.6	0.1	2.6	1.7		1.4	1.4
**I3.1**	Drug changed to …	0.8		1.1		2.5		1.4	2.7	1.9	
**I3.2**	Dosage changed to …		0.1	0.8				0.7	0.6	0.9	
**I3.3**	**Formulation changed to …**	1.2		0.1	0.2	0.4		29.0			0.7
**I3.4**	**Instructions for use changed to …**	0.1	0.8	0.1		0.1	0.3	13.1	0.1	1.4	
**I3.5**	Drug paused or stopped			0.4	0.2	0.3		0.1		0.5	
**I3.6**	**Drug started**	0.2				3.3		2.8	43.7		
**I4.1**	Other intervention	0.1	0.1	0.1	0.3			0.1			0.7
**Non-codable intervention**		6.2	4.8	8.9	6.1	5.5	1.7	3.0	7.6	14.6	29.6

Figure E2: Prevalence (in percentages) of identified interventions for drug-related problems in each e-learning module. The darker the color, the more frequently the intervention was performed in the respective module. Only interventions with at least one identified case are displayed, as well as percentages >0.0%.
